# Pitfalls in diagnosis of infiltrative lung disease by CT

**DOI:** 10.1259/bjro.20190036

**Published:** 2019-10-01

**Authors:** Céline Du Pasquier, Rami Hajri, Romain Lazor, Cécile Daccord, Stacey Gidoin, Michel Brauner, Catherine Beigelman-Aubry

**Affiliations:** 1 Department of Radiology, Lausanne University Hospital and University of Lausanne, Lausanne, Switzerland; 2 Respiratory Medicine Department, Lausanne University Hospital and University of Lausanne, Lausanne, Switzerland; 3 Department of Radiology, Université Paris Nord 13, Hôpital Avicenne, Bobigny, France

## Abstract

The diagnosis of interstitial lung disease may be challenging, especially in atypical disease. Various factors must be considered when performing and reading a chest CT examination for interstitial lung disease, because each of them may represent a source of misinterpretation. Firstly, technical aspects must be mastered, including acquisition and reconstruction parameters as well as post-processing. Secondly, mistakes in interpretation related to the inaccurate description of predominant features, potentially leading to false-positive findings, as well as satisfaction of search must be avoided. In all cases, clinical context, coexisting chest abnormalities and previous examinations must be integrated into the analysis to suggest the most appropriate differential diagnosis.

## Introduction

Currently, high-resolution CT (HRCT) of the chest is the imaging modality of choice in the diagnostic process of interstitial lung diseases (ILDs), as its findings have a major impact on clinical management, including decisions regarding a specific therapy or less commonly the need for lung biopsy. Therefore, an accurate radiological assessment is of greatest importance in terms of diagnosis, prognosis and therapeutic management.^1,2^ Radiologists and clinicians must be aware of potential sources of error and know how to avoid them.

### Technical factors

#### Body position

**Figure 1.**
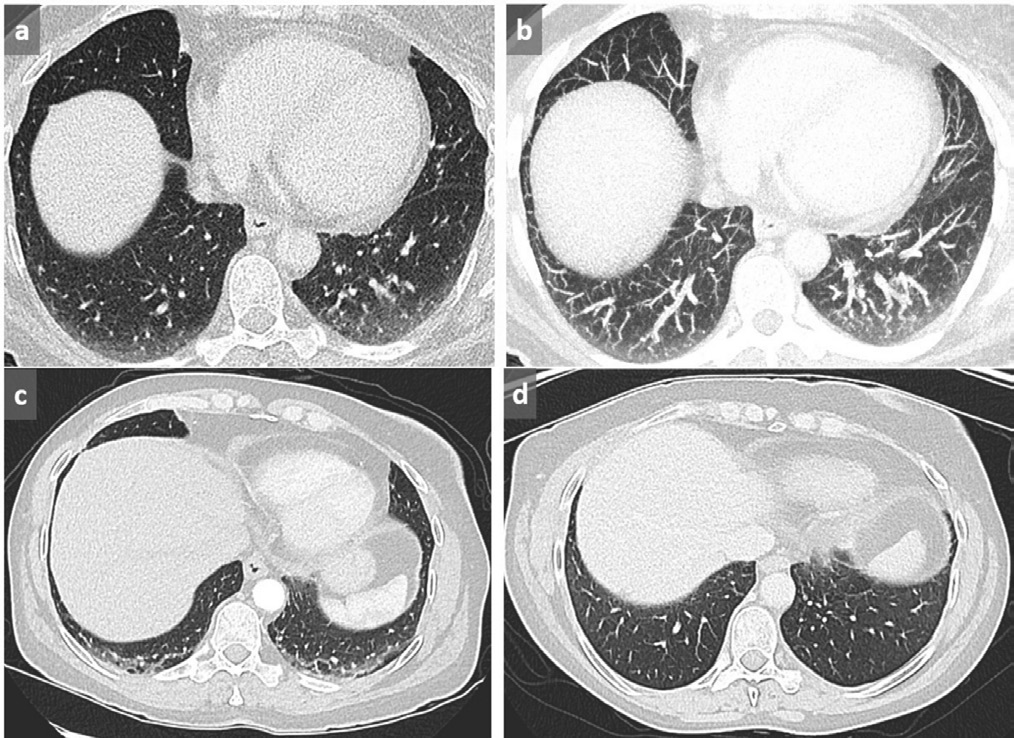
Dependent GGO. Increased lung attenuation in subpleural and basal location in dependent areas may be favored by lack of inspiration (a). By using MIP, tortuous vessels in high attenuating areas are clearly identified, confirming the lack of deep inspiration (b). In another case of a 58-year-old patient presenting with shortness of breath, cough and hemoptoic sputum, supine acquisition shows bilateral GGOs in a posterobasal location with relative sparing of the immediate subpleural area suggestive of NSIP (c). This diagnosis is excluded by the reversibility of these dependent abnormalities in prone position (d). GGO, ground glass opacity; MIP, maximum intensityprojection; NSIP, non-specific interstitial pneumonia.

**Figure 2.**
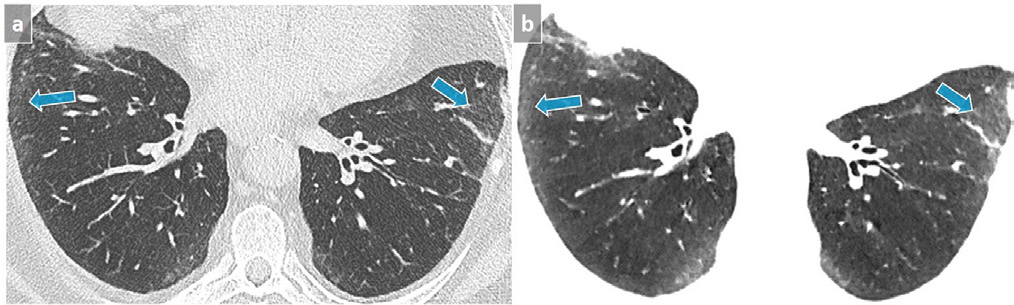
NSIP. Subpleural GGO in a posterobasal and subpleural location that could suggest dependent abnormalities and require another acquisition in prone position. However, the presence of a subtle GGO in subpleural lateral areas (arrows) precludes the need for an additional prone acquisition (a). A 3 mm-thick mIP slice increases our diagnostic confidence by reinforcing the visibility of abnormal densities (b). GGO, ground glass opacity; mIP, minimum intensity projection.

Most CT scans are performed in supine position, and it is well known that dependent ground glass opacities (GGO) may occur, especially when inspiration is suboptimal. In this case, the tortuosity of the vessels, well depicted using maximum intensity projection (MIP), confirms the lack of deep inspiration (Figure 1). In order to exclude true parenchymal abnormalities, an additional acquisition in prone position focused on abnormal areas is required, most commonly on lung bases (Figure 1). Importantly, the presence of similar findings in non-dependent zones definitely confirms the real nature of the abnormalities. In this setting, additional acquisition is not needed, thus avoiding unnecessary radiation exposure (Figure 2).

In order to avoid such anomalies, the whole CT acquisition may be performed directly in prone position in specific settings, such as systemic sclerosis, a condition associated with nonspecific interstitial pneumonia which consists of GGO and reticulations with a typical basal and subpleural distribution.^3^ Such a strategy reinforces diagnostic confidence in case of posterobasal abnormalities while minimizing radiation exposure.

**Figure 3.**
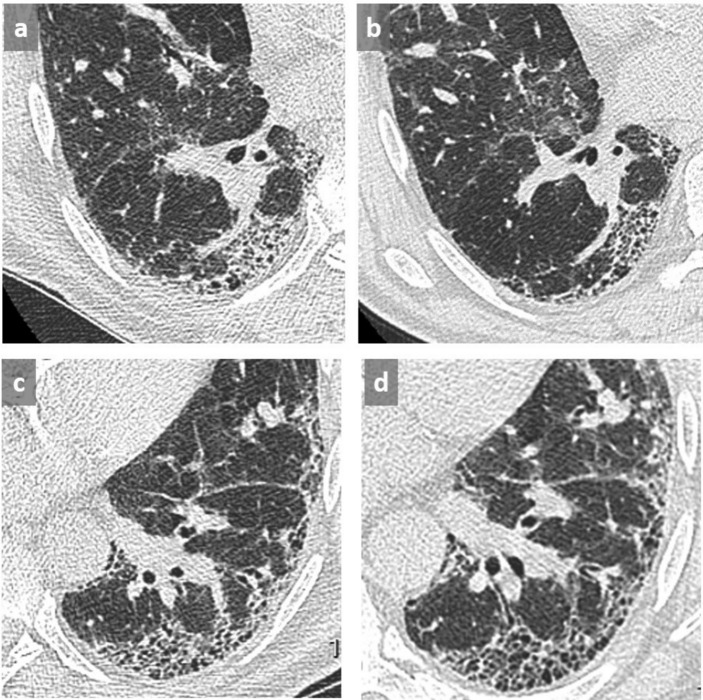
Thin slices at the level of right lower lobe (a, b) and left lower lobe (c, d) in a patient with UIP in supine (a, c) and prone (b, d) position. Identification of the honeycombing pattern in posterobasal location appears difficult in supine position due to superimposed GGO and lack of lung expansion (a, c) conversely to prone position (b, d) where the diagnosis of honeycombing is facilitated, allowing a definite diagnosis of a UIP pattern. GGO, ground glass opacity; UIP, usual interstitial pneumonitis.

Moreover, significant differences may be observed between supine and prone acquisitions, which may alter the final diagnosis (Figure 3). To avoid potential pitfalls, the most recent classification of idiopathic interstitial pneumonias recommends performing both supine and prone acquisitions, the latter being systematic or optional.^2,4^ To allow for a reliable comparison, follow-up studies should be performed in the same position than that of the initial evaluation.

### Dose parameters

**Figure 4.**
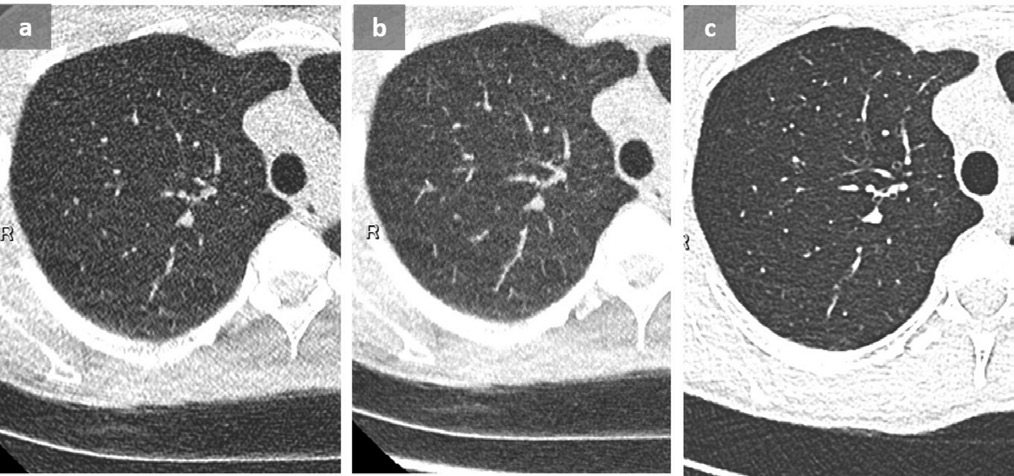
When performing an acquisition with a very low CTDI at 0.11 mGy, image noise, well seen outside of the chest, can mimic a miliary disease on thin axial slice (a) and MIP reformat (b), even though applying an iterative reconstruction algorithm. A follow-up CT with a CTDI at 0.29 mGy (b) reduces noise, allowing to exclude micronodules. These CT were performed in a context of recurrent pneumothorax in a young patient with endometriosis (not shown). CTDI, CT dose index; MIP, maximum intensity projection.

**Figure 5.**
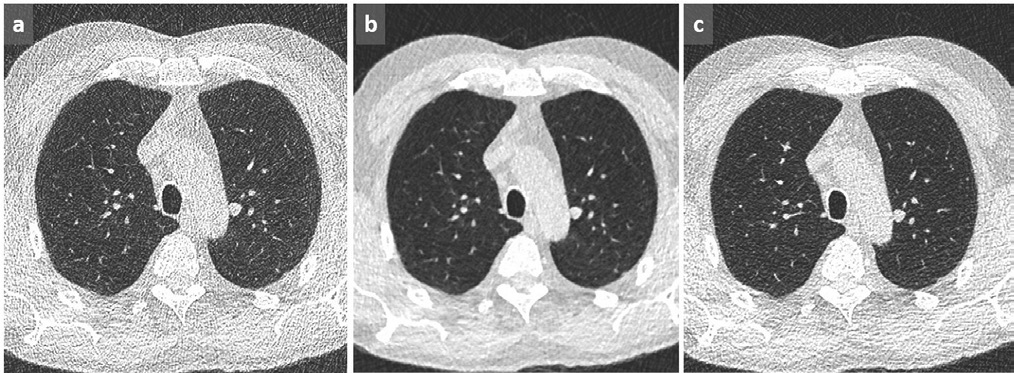
Lung CT acquisition with a CTDI at 0.45 mGy and DLP at 14 mGyxcm with lung (a), soft (b) and intermediate (c) kernel shows an optimized balance between image noise and spatial resolution with the intermediate filter at the same dose. CTDI, CT dose index; DLP, dose–length product.

According to Raghu,^2^ reduced doses [otherwise named low dose (LD)] between 1 and 3 milliSieverts (mSv) are recommended for the assessment of ILDs. This may be achieved by selecting an appropriate CT dose index depending on the latest iterative reconstruction (IR) algorithms available, this combined with tube current modulation. Conversely, ultra-low doses (<1 mSv) (ULD) are inappropriate in this setting, as they may be a source of misdiagnoses due to lack of detection or misleading interpretation of abnormal findings. In addition, by increasing the image noise, ULD acquisition may mimic disorders such as miliary disease despite the use of IR algorithms (Figure 4). To minimize these potential drawbacks, the best approach when using LD CT consists in finding the best kernel compromise (Figure 5) to ensure an optimal balance between spatial resolution and image noise.

### Reconstruction parameters

**Figure 6.**
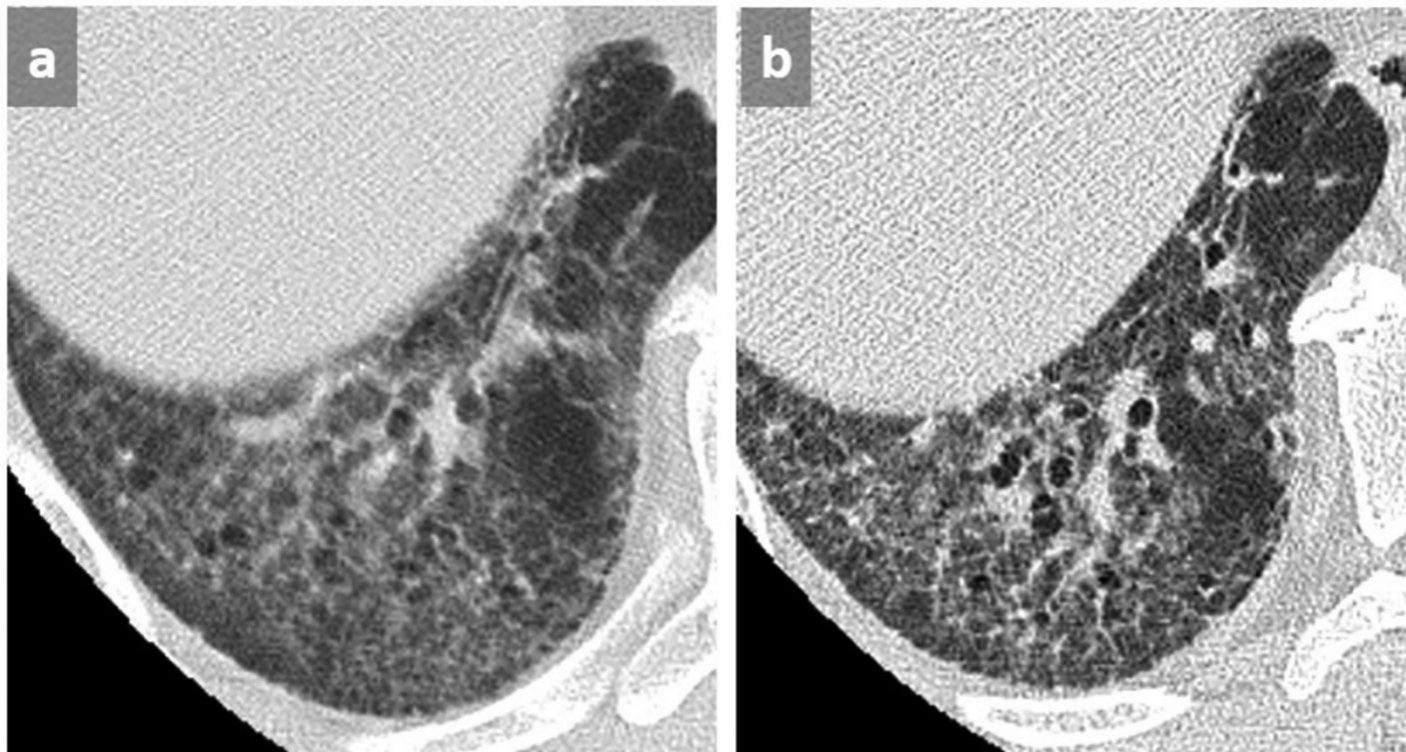
Interstitial lung disease in systemic sclerosis. On the left, subtle details such as intralobular reticulations are completely missed due to partial volume effect with thick slices 5 mm-thick (a). For this reason, thin slices, 1.25 mm-thick in this case, must always be used (b).

Reconstruction with thin slices at best overlapped is essential to avoid partial volume effect, which precludes the analysis of subtle abnormalities such as intralobular reticulations (Figure 6). In the same time, this ensures an adequate quality of reformats in any plane, whether coronal, sagittal or in the long axis of bronchi, which may help to recognize traction bronchiectasis/bronchiolectasis faced with cystic lesions or subpleural reticulations, a key feature for the diagnosis of usual interstitial pneumonitis.

**Figure 7.**
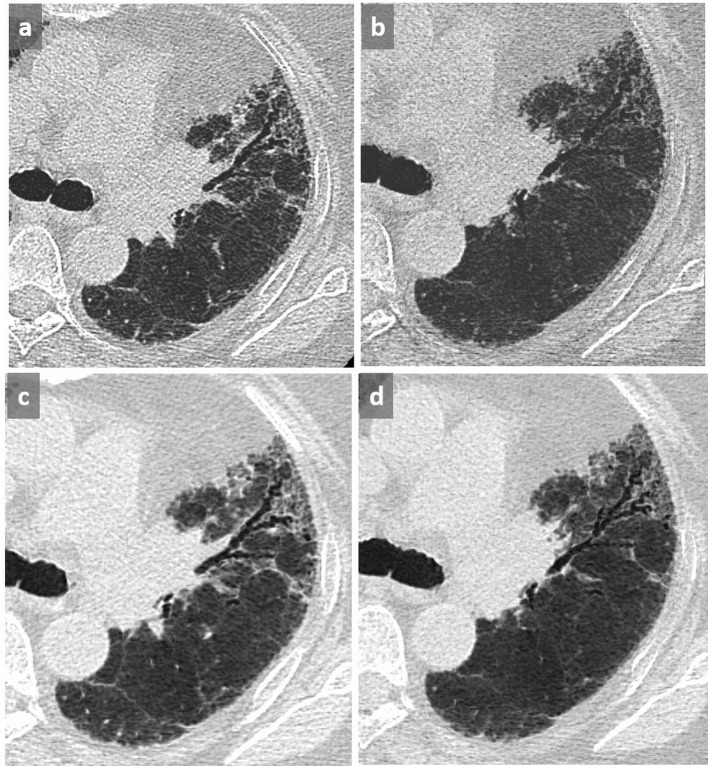
Optimal rendering of mIP by using the soft kernel compared to the lung one. (a) mIP 3.3 mm with lung kernel; (b) mIP 6.5 mm with lung kernel; (c) mIP 3.3 mm with soft kernel; (d) mIP 6.5 mm with soft kernel. Whatever the slab thickness of the mIP post-processing tool, there is a lower image quality by using the lung kernel compared with the soft one. Assessment of GGO and traction bronchiectasis is much better depicted in (c, d). GGO, ground glass opacity; mIP, minimum intensity projection.

Furthermore, an adequate choice of kernel reconstruction is required for optimal rendering with post-processing tools. In particular, minimum intensity projection (mIP) used with lung kernel is commonly associated with inadequate image quality, especially at low dose. To restore image quality, it is relevant to apply it on reconstructions with soft tissue kernel and lung windowing (Figure 7).^5^ As a recall, mIP post-processing tool that displays the lowest attenuation value of a voxel throughout a volume is aimed at offering an optimal detection of GGO (Figure 2), together with an excellent assessment of distal bronchiectasis/bronchiolectasis as well as areas of decreased attenuation.

### Interpretation pitfalls

#### Anomalies without clinical significance

**Figure 8.**
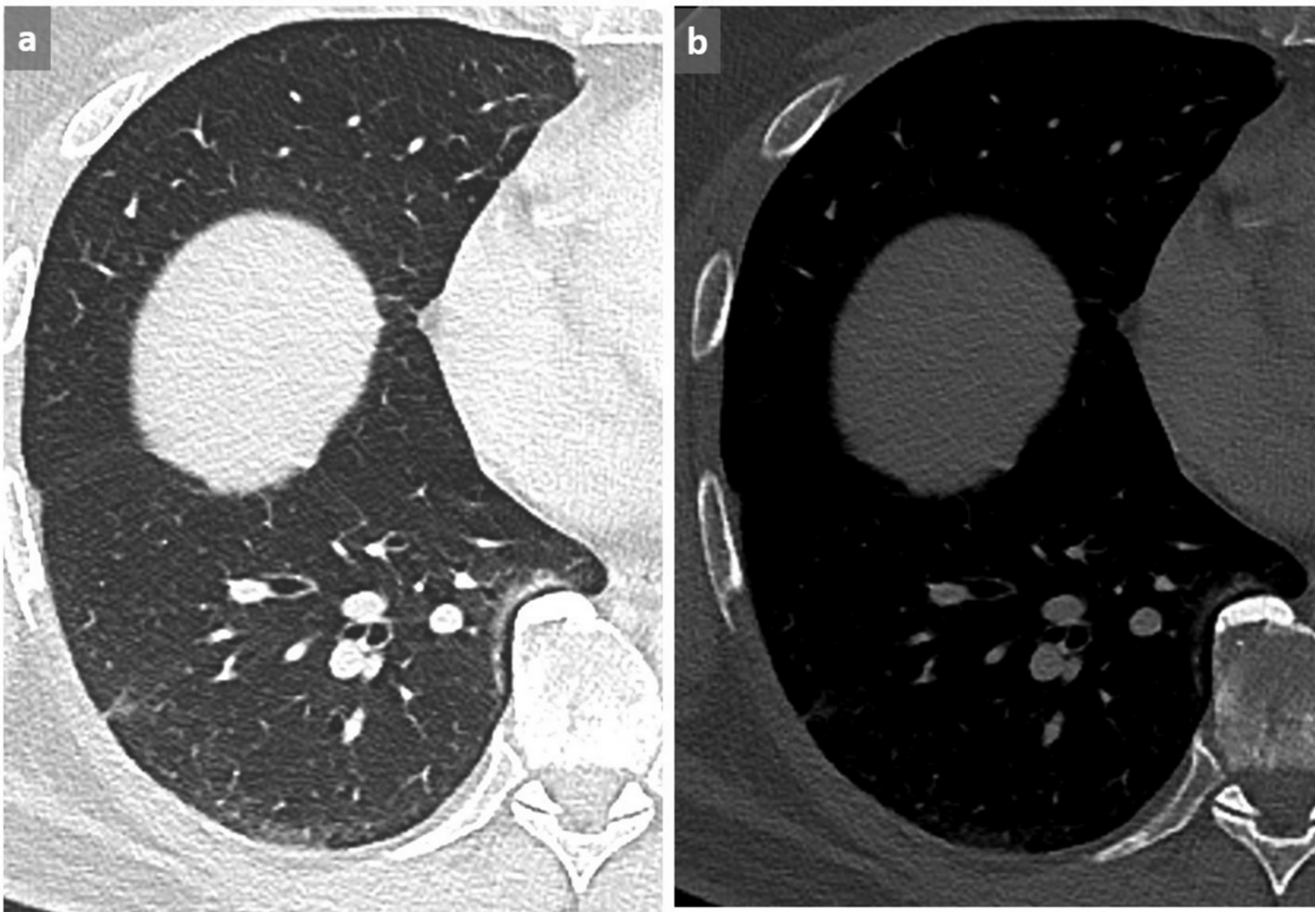
Paravertebral GGO (a) near a protruding osteophyte (b) must not be considered as abnormal and therefore should not be reported as pathological. Note dependent GGO in subpleural area that were reversible in prone position. GGO, ground glass opacity.

Linear opacities or GGO in the immediate vicinity of protruding structures like osteophytes, are not pathologic and should not be reported as such. (Figure 8).

### Micronodules

**Figure 9.**
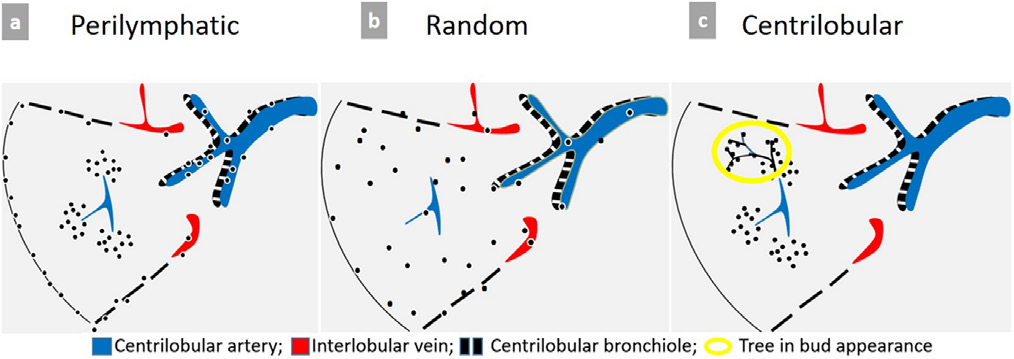
Schematic representation of micronodules distribution patterns. (a) Typical perilymphatic distribution involving the parahilar peribronchovascular interstitium until terminal bronchioles, the subpleural area, along the fissures or interlobular septa. A typical perilymphatic distribution is seen in sarcoidosis, lymphangitic carcinomatosis or silicosis. (b) In case of random distribution, the distribution is uniform without respect of anatomic structures. This suggests a hematogenous spread of disease, particularly miliary metastases, tuberculosis, fungal or viral infection. (c) Centrilobular distribution is characterized by the presence of multiple small nodules often ill-defined grouped within the center of the secondary pulmonary lobule, with a location at least 3 mm away from the pleura. Therefore, the key point for the recognition of this pattern is the absence of any nodule along the pleural interface. This distribution is primarily suggestive of bronchial and peribronchial disease, but may also be related to vascular or perivascular disease, and more rarely interstitial disease predominating around the centrilobular bronchiole and artery.

MIP post-processing is the optimal tool required for the detection and accurate description of nodules and micronodules, whose distribution can be perilymphatic, random (miliary), or centrilobular (Figure 9).

**Figure 10.**
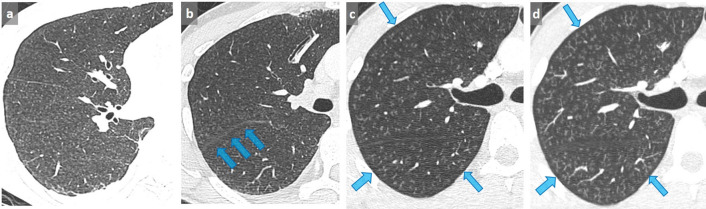
False-positive diagnosis of miliary disease. Faced with this pattern of profuse micronodules (a), this patient was diagnosed with miliary tuberculosis, and consequently treated with antituberculous quadritherapy. However, the patient experienced a worsening of dyspnea and severe cough when he returned back home. The final diagnosis was hypersensitivity pneumonitis related to a humidifier use. Note the sparing of the juxtafissural area, which allows a definite diagnosis of centrilobular nodules (arrows) (b). A miliary disease was initially diagnosed on thin slices in this other patient with severe dyspnea and hypoxemia (c). A 4 mm-thick MIP demonstrates a tree-in-bud appearance (d), with a typical sparing of the subpleural area characteristic of centrilobular nodules, more difficult to assess on thin section. In association with the tree in bud appearance, this was strongly suggestive of bronchiolitis that was related to cannabis exposure and subsequently resolved after interruption of its use. MIP, maximum intensity projection.

Some pitfalls may be encountered. For example, the distribution of micronodules in sarcoidosis is typically perilymphatic. However, in case of profuse micronodular infiltration, its perilymphatic distribution may be difficult to recognize and may simulate a miliary disease. Similarly, when it comes to distinguishing between centrilobular and miliary pattern of distribution, it is recommended to pay particular attention to juxtafissural areas. The absence of nodules near the pulmonary fissures excludes the diagnosis of miliary disease (Figure 10).

### Tree-in-bud pattern

Sometimes, centrilobular micronodules are connected with small branching linear opacities corresponding to upstream thickened or filled dilated bronchioles, at best demonstrated with MIP post-processing tool. This tree-in-bud pattern helps to categorize multiple nodules on thin slices (Figures 9–10). Although most often related to infectious or inflammatory bronchiolitis, tree-in-bud appearance may also reflect pulmonary arterial metastasis.^6^

### Honeycombing *vs* pseudohoneycombing

Paraseptal emphysema corresponding to a predominant destruction of the distal alveoli and their ducts and sacs^7^ appears as well-marginated hypodensities with distinct walls corresponding to septa and arranged in one layer, without associated features of fibrosis. Conversely, honeycombing typically manifests as multiple layers of cystic airspaces with thick walls, together with other signs of fibrosis, including traction bronchiectasis, irregular reticulations and volume loss. These two entities may be associated in the combined emphysema-fibrosis syndrome, and it may be difficult even impossible to differentiate emphysema from honeycombing, despite the use of mIP in oblique reformats (see "Reconstruction parameters").

**Figure 11.**
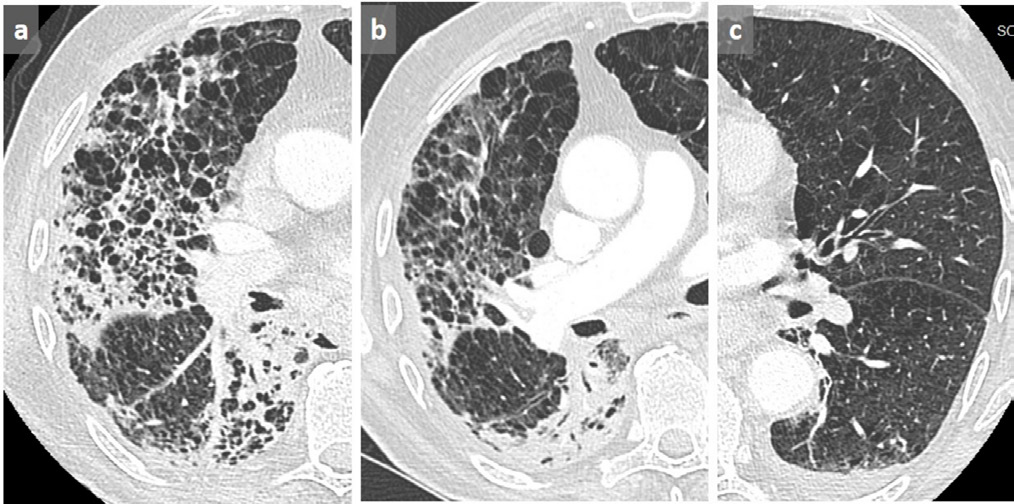
Pseudohoneycombing. Patient known for COPD presenting with cough. Honeycombing was reported on the first CT scan (a). Subsequent axial chest CT 2 months later showed disappearance of the cystic pattern that was related to a resolution of alveolar condensation after antibiotic therapy for *S.* *pneumoniae* infection (b). Note the controlateral emphysematous changes related to COPD (c). COPD, chronic obstructive pulmonary disease.

There is a common overdiagnosis of honeycombing in patients with chronic obstructive pulmonary disease. Indeed, the filling of alveoli surrounding the emphysematous changes may mimic honeycomb pattern. This is generally observed in case of superimposed infection (Figure 11) or alveolar hemorrhage. The presence of emphysematous changes in the other lung and the disappearance of the pseudohoneycomb changes after treatment allow avoiding this potentially harmful pitfall.

### Mosaic ground glass opacities

**Figure 12.**
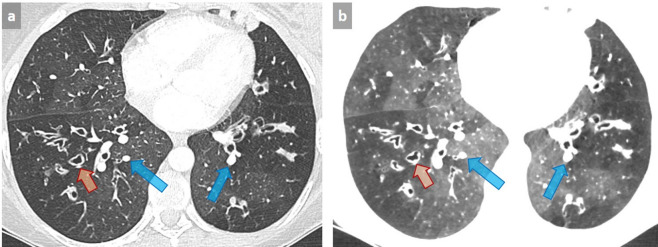
Patient with cystic fibrosis with mosaic perfusion pattern related to small airway disease. Mosaic perfusion pattern appears as abnormal areas of decreased attenuation associated with small vessel size (orange arrows) alternating with preserved areas in which larger vessels respond to an increase in arterial blood flow (blue arrows) (a). Note the improved visibility of normal and abnormal areas by using 4 mm-thick mIP post-processing with lowering of the window level and reduction of the window width (−820, 572 HU) (b). Such an aspect should not be confused with GGO with mosaic appearance, in which areas of GGO are the abnormal zones. GGO, ground glass opacity; HU, Hounsfield unit; mIP, minimum intensity projection.

**Figure 13.**
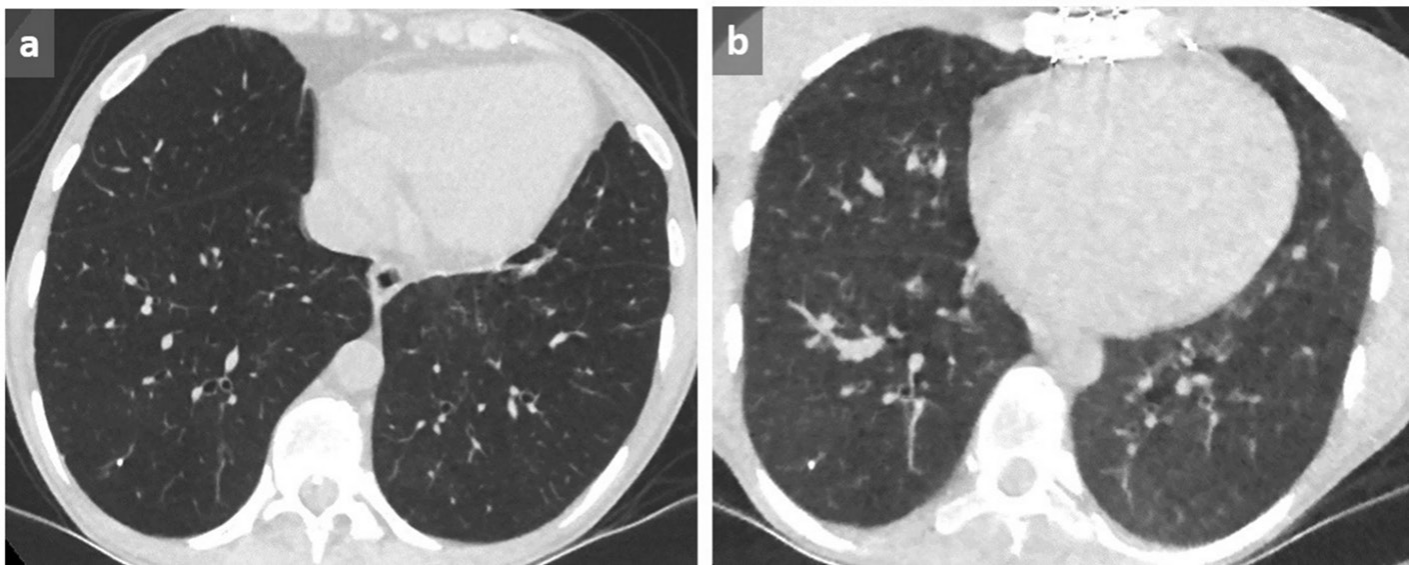
Post-transplant diffuse constrictive bronchiolitis. Axial chest CT of a post-transplant 31-year-old female showing diffuse decreased attenuation of the lung parenchyma in inspiration (a) without visible air trapping on expiration (b). A severe constrictive bronchiolitis was confirmed histologically.

Some imaging patterns can mimic ILD and lead to erroneous diagnosis. A classical example is mosaic appearance, in which the diagnostic approach is based on the assessment of the caliber of the vessels in black and gray areas. Typically, an equivalent vessel caliber in both black (decreased attenuation) and grey (increased attenuation) areas correspond to GGO with mosaic appearance, with areas of GGO being the abnormal zones. Conversely, when vessels within regions with decreased attenuation appear smaller than in areas with increased attenuation, this corresponds to a mosaic perfusion pattern, related to regional decreases in lung perfusion. In this case, areas with decreased attenuation are the pathologic zones, and GGO appearance results from the increase in capillary blood flow as a consequence of vascular redistribution in the normal lung, thus leading to a geographic appearance (Figure 12). Mosaic perfusion pattern may be related either to airway or vascular disease, the latter being classically observed in chronic pulmonary embolism, typically associated with an enlargement of pulmonary trunk and/or right heart chambers. In case of mosaic pattern of bronchial and/or bronchiolar origin, the presence of abnormal airways with parietal wall thickening or bronchiectasis is suggestive of the diagnosis. It can be confirmed by air trapping on expiration, whose quality is attested by anterior bowing of the posterior tracheal membrane. However, some cases such as severe constrictive bronchiolitis after transplantation may represent a pitfall, as no significant change is observed between expiratory and inspiratory images (Figure 13). Finally, i.v. contrast can accentuate mosaic attenuation.

### Acute exacerbation of interstitial lung disease

**Figure 14.**
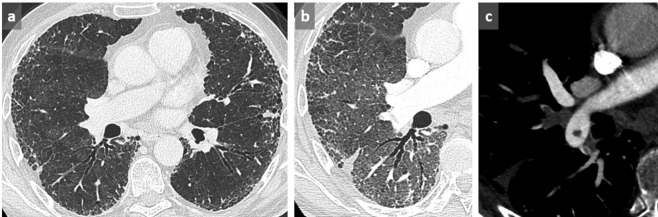
Pulmonary embolism in a patient with suspected acute exacerbation of interstitial lung disease. 73-year-old male known for drug-induced pulmonary fibrosis (a) (chemotherapy for high-grade bladder carcinoma), presenting with increasing dyspnea, fever and oxygen desaturation. Although a new GGO was observed and attributed to fibrosis exacerbation (b), pulmonary emboli were detected on this contrast-enhanced chest CT (c). Note the triangular opacity corresponding to a small intrafissural effusion in (b). This example highlights the importance of performing a contrast-enhanced CT in case of clinical worsening in patients with pulmonary fibrosis to rule out pulmonary embolism, since acute exacerbation is a diagnosis of exclusion. GGO, ground glass opacity.

In case of acute or subacute clinical deterioration with new GGO areas on CT scan, acute exacerbation of the known ILD is a diagnosis of exclusion, and it is essential to rule out superimposed infection, drug-induced pneumonitis, pulmonary edema, as well as pulmonary embolism by contrast-enhanced angio-CT. (Figure 14).

### Missed lung cancers

**Figure 15.**
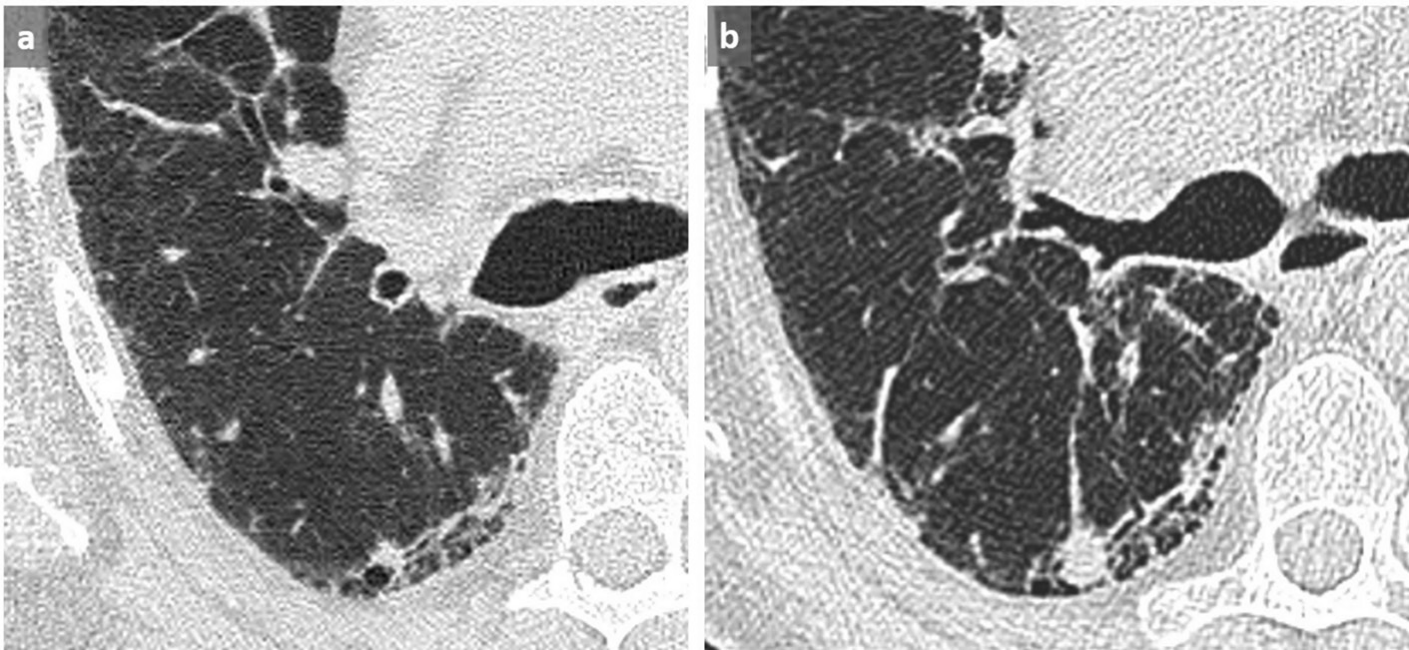
Missed cancer in a patient known for UIP. A non-specific nodule/density at the frontier of the diseased/normal lung was not reported on the first CT scan (a). A significant increased size was subsequently observed at the follow-up CT scan 5 months later (b) with a histologically proven low differentiated lung carcinoma. Satisfaction of search which is mainly aimed at evaluating the ILD commonly overlook such focal and/or newly discovered suspicious abnormalities. ILD, interstitial lung disease; UIP, usual interstitial pneumonitis.

Lung cancers in a patient known for UIP are commonly observed and may present as a non-specific nodule or density in all lung areas, including at the frontier of the diseased fibrotic and normal lung.^8,9^ Such abnormalities that should be considered as suspicious in this context, especially if newly appeared or if they show significant growing, are commonly overlooked especially due to the concept of satisfaction of search^10^ (Figure 15), leading the radiologist/pulmonologist to only focus on the ILD by itself, whether to characterize or to evaluate its evolution. A careful analysis of the whole lung parenchyma should allow avoiding this potentially harmful pitfall.

## Conclusions

An accurate interpretation of imaging in ILD requires good knowledge of the potential pitfalls related to body position and technical parameters. To allow an optimal diagnosis and management, it is recommended to follow a systematic approach based on the identification of the predominant abnormal pattern, which requires the knowledge of anatomical structures and mimickers, which may occur in this setting. Finally, focal anomalies should be carefully considered in the setting of UIP.
